# The Histamine Four Receptor Regulates LFA-1 But Not Mac-1 -Dependent Neutrophil Adhesion and Inflammatory Functions

**DOI:** 10.1007/s10753-026-02508-4

**Published:** 2026-04-27

**Authors:** Karim Dib

**Affiliations:** 1 Wellcome-Wolfson Institute for Experimental Medicine, Queen’s University Belfast, Belfast, Northern Ireland UK; 2 Faculty of Medicine, Frankfurt Branch, European University Cyprus, Frankfurt am Main, Germany; 3 School of Life and Health Sciences, Frankfurt Branch, European University Cyprus, Frankfurt am Main, Germany

**Keywords:** Neutrophils, Histamine, Small GTPases, Histamine four receptor

## Abstract

Neutrophils incubated on ICAM-1- or fibrinogen- coated plates produced histamine in response to fMLP indicating that histamine may be an intermediary chemoattractant promoting β2 integrin-dependent adhesion. fMLP, but not histamine or the histamine four receptor (H_4_R) agonist VUF 8430, augmented adhesion of neutrophils, or neutrophil-like PLB-985 cells, to fibrinogen, a Mac-1 ligand. This result may be explained by the fact that fMLP, but not histamine, augmented intracellular calcium, a pre-requisite for Mac-1 expression on the cell surface. fMLP- induced Mac-1- dependent adhesion was not affected by histamine or VUF 8430. fMLP induces conformational activation of LFA-1, but neutrophil adhesion to ICAM-1 was not augmented by fMLP. Histamine did not augment neutrophil adhesion to ICAM-1, as histamine does not induce conformational activation of LFA-1. Importantly, fMLP augmented neutrophil adhesion to ICAM-1 when combined with histamine or VUF 8430. Thus, activation of the H_4_R and the formyl peptide receptor 1 may be required to augment LFA-1 avidity. Histamine activated Rap1, Rac1/2 and Cdc42 in neutrophil-like PLB-985 cells. These effects were blocked by Src tyrosine kinase inhibitors (for Rap1 and Rac1/2 but not Cdc42). VUF 8430 activated these GTPases in PLB-985 cells and neutrophils indicating that the H_4_R controls neutrophil functions. Intriguingly, histamine inhibited fMLP-induced specific granule but not azurophil granule exocytosis by adherent neutrophils. Histaminase is present in specific granules. Therefore, inhibition of specific granule exocytosis may be a mechanism to limit the catabolism of histamine produced by adherent neutrophils to allow H_4_R-mediated inflammation through activation of monomeric GTPases and LFA-1.

**Graphical Abstract:**

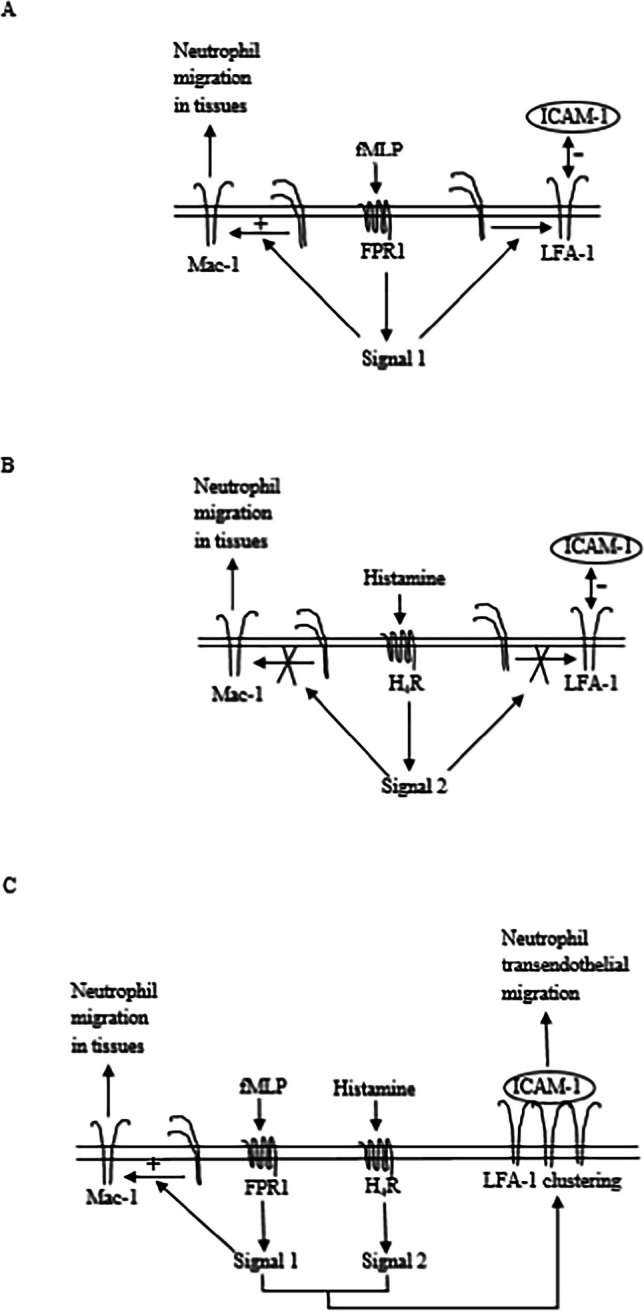

## Introduction

Neutrophils migrate to infected tissues and activate a broad range of antimicrobial functions to eliminate pathogens. These events are controlled by soluble mediators produced during the inflammatory response. These include TNF-α formed by tissue-resident macrophages, primary chemoattractants such as formyl-peptides synthesized by bacteria, and complement 5a produced through activation of the complement cascades [1].

A common feature of soluble mediators of inflammation is their ability to induce translocation of Mac-1-containing granules to the cell membrane [2] and activation of the β2 integrins Mac-1 (CD11b/CD18) and LFA-1 (CD11a/CD18). This is achieved through conformational change of the β2 integrins (affinity alteration) from a low to a high affinity ligand binding conformation [3, 4]. The switch from a low affinity bent to a high affinity extended conformation can be monitored using monoclonal antibodies binding specifically to the active extended forms of Mac-1 (CBRM1/2) [3] or LFA-1 (KIM127 and NKI-L16, mAb24) [4]. Another means of β2 integrin activation is through avidity alteration which involves organization of β2 integrins into clusters on the cell surface leading to augmented valency [5–8]. Affinity and avidity alterations are critical elements of neutrophil adhesion to the vascular endothelium, migration into inflamed tissues and activation of neutrophil inflammatory functions.

Conformational change of β2 integrins in response to soluble ligands is tightly dependent on activation of the small GTPase Rap1. This is well documented by the work of Caron et al. [9] showing that expression of a constitutively active form of Rap1 in macrophages is sufficient for functional activation of Mac-1 and binding of complement-opsonized particles to this integrin. Rap1 is also part of the multi-subunit enzyme NADPH oxidase through its interaction with cytochrome b (p22^phox^) [10]. Evidence for a role of Rap1 in the production of reactive oxygen species (ROS) is best exemplified by studies showing that production of superoxide in response to fMLP is reduced in neutrophils from Rap1a^−/−^ mice [11].

The small GTPases of the Rho family play a key role in the migration of neutrophils to the site of infection. Cell motility depends on the dynamic formation of membrane protrusions such as lamellipodia (driven by Rac GTPases) and filopodia (driven by Cdc42) at the leading edge of migrating leukocytes [12]. Formation of these protrusions is dynamic and involves Rac-dependent actin assembly and disassembly via uncapping of actin filaments [13].

In addition to their involvement in regulating cell motility, Rac and Cdc42 regulate neutrophil inflammatory functions. Indeed, Rac GTPases are part of a functional NADPH oxidase complex which produces ROS [14]. Rac and Cdc42 also control the fusion of neutrophil granules with the phagosome, via a mechanism dependent on the MAPK pathway, to target ingested pathogens [15].

A hallmark of small GTPases is their ability to cycle between a GDP-bound inactive to a GTP-bound active form. Guanine nucleotide exchange factors regulate the GDP-GTP switch. When the GTPases are loaded with GTP, they interact with effectors to initiate downstream signaling and functions [16].

The recruitment of neutrophils to the site of inflammation is further amplified by soluble intermediary mediators produced by neutrophils which act as autocrine/paracrine signals. Thus, interaction of neutrophils with *Salmonella typhimurium* increased leukotriene B4 (LTB_4_) production in response to fMLP and hydrogen sulfide, a gas produced by bacteria [17]. Gradient of LTB_4_ concentrations, through activation of the BLT1 receptor, drive neutrophil recruitment and swarming to sites of tissue damage and infection [18, 19]. Neutrophils also produce the chemokine IL-8 in response to fMLP, C5a, or platelet-activating factor (PAF) to extend chemotactic and inflammatory responses [20]. A sustained production of IL-8 by neutrophils made adherent by Mac-1 has also been reported [21].

Neutrophils synthesize and release histamine which contributes to airways inflammation in mycoplasma pneumonia [22] or during a neutrophil-mediated LPS-induced model of acute lung injury [23]. It is not known whether histamine is produced in response to primary chemotactic signals and therefore whether it can act, similarly to IL-8 and LTB_4_, as a secondary mediator.

Neutrophils express two histamine receptors: the histamine two receptor (H_2_R) and the histamine four receptor (H_4_R) [24]. The H_4_R has a high affinity for histamine and is activated by nanomolar concentrations of histamine. In contrast the H_2_R has a low affinity for histamine and is activated by micromolar concentrations of the diamine [25].

There is evidence in the literature showing that the H_2_R has anti-inflammatory functions. Histamine inhibits neutrophil spreading and Mac-1 clustering [26]. Concentrations of histamine in the micromolar range inhibit fMLP-induced hydrogen peroxide production, release of β-glucuronidase [27] and leukotriene biosynthesis [28]. These effects of histamine were prevented by H_2_R antagonists [28] or mimicked by H_1_R/H_2_R agonists [27]. Histamine at a concentration of 10^–6^ M has also been shown to inhibit the capture by neutrophils of complement opsonized *E. coli* [24].

There is paucity of information on the role played by the H_4_R in the regulation of neutrophil functions. We showed that engagement of the H_4_R in neutrophils led to inhibition of adhesion-dependent exocytosis of specific granules [29] indicating that the H_4_R has anti-inflammatory functions. However, we also found that engagement of the H_4_R in neutrophils accelerated the killing of engulfed *E. coli* [24], a process which requires production of ROS and fusion of granules containing antimicrobials with phagosomes.

In the present study, we aimed at investigating: 1) Mac-1- and LFA-1- dependent production of histamine by adherent human neutrophils; 2) The role played by the H_4_R in the regulation of neutrophil adhesion and inflammatory functions.

To this end, we studied the regulation of Rap1, Rac1/2 and Cdc42 in response to histamine and the H_4_R agonist VUF 8430 [30]. The rationale for using these readouts is that these monomeric GTPases control neutrophil inflammatory functions as well as β2 integrin-dependent adhesion and migration.

Finally, we compared the effect of histamine on exocytosis of specific and azurophil neutrophil granules to understand the biological meaning of the inhibition by histamine of specific granule exocytosis by adherent neutrophils.

## Materials and Methods

### Materials

The histamine ELISA kit was from IBL International GmbH/TECAN (Hamburg, Germany). Histamine (Ref: H7125), VUF 8430 (Ref: V4390), fMLP (Ref: F3506), human lactoferrin (Ref: L0520), anti-lactoferrin Abs (Ref: L3262), fibrinogen from human plasma (Ref: F3879), and SU 6656 (Ref: 572635) were purchased from Merck Life Science (UK).

The Src tyrosine kinase inhibitor PP2 (ab120308), and human recombinant ICAM-1 (ab168688) were from Abcam (Cambridge, UK).

Ficoll-Hypaque was from GE Healthcare/Cytiva and Dextran 500 from Pharmacosmos (Denmark). For tissue culture, RPMI 1640 medium, antibiotics (penicillin/streptomycin) (Ref: 15140122), and fetal bovine serum (A5256701) were from Thermo Fischer scientific (UK).

The following antibodies were from Cell Signaling: the secondary HRP-conjugated anti-rabbit antibodies (7076S); rabbit anti-Rac1/3 (#2465); anti-Rap1A/Rap1B (#2399), and anti-Cdc42 (#2462) antibodies.

### Methods

#### Isolation of Human Neutrophils

Venous blood was collected from healthy donors by venous puncture in vacutainer EDTA blood collecting tubes. Neutrophils were isolated from the blood using Dextran sedimentation and centrifugation through Ficoll-Hypaque [24]. The cells (97% purity) were resuspended in HBSS medium supplemented with 20 mM Hepes; pH 7.4, 1 mM CaCl_2_ and 1 mM MgCl_2_ (modified HBSS) at a concentration of 0.5 × 10^6^ cells/ml (for adhesion assays) or 10^7^ cells/ml (for GTPases assays).

#### Cell Culture

Undifferentiated PLB-985 cells were grown at 37 °C in an atmosphere of 5% CO_2_ in RPMI medium supplemented with 10% FCS to a density of 5 × 10^5^cells/ml. Differentiation into neutrophil-like cells was carried out by culturing undifferentiated PLB-985 cells for 5 days in RPMI medium supplemented with 5% FCS and 1.25% DMSO [31]. Cells were then collected by centrifugation (190 g, 10 min), washed in HBSS medium devoid of magnesium, calcium and phenol red (Ref: 14175095) supplemented with 20 mM Hepes; pH 7.4, and re-suspended in the same medium. After one hour at 37 °C in a 50 ml tube, the cells were re-suspended at a density of 0.5 × 10^6^ cells/ml or 10^7^ cell/ml in modified HBSS medium.

#### Adhesion Assays

Easy Grip™ Petri dishes were incubated overnight at 4 °C with either fibrinogen (20 μg/ml) or human recombinant ICAM-1 (0.5 μg/ml) both dissolved in modified HBSS. Neutrophils (0.5 × 10^6^) were allowed to adhere to fibrinogen- or ICAM-1- coated dishes at 37 °C in the presence of fMLP (10 ^−6^ M), histamine (10 ^−9^ M- 10 ^−6^ M), VUF 8430 (10 ^−9^ M- 10 ^−6^ M) or a combination of fMLP (10 ^−6^ M) and histamine (10 ^−9^ M- 10 ^−6^ M) or a combination of fMLP (10 ^−6^ M) and VUF 8430 (10 ^−9^ M- 10 ^−6^ M). After 30 min, non-attached cells were removed by aspiration, and plates were washed three times with sterile PBS. Adherent cells were fixed for 30 min with 3.4% paraformaldehyde at room temperature. Thereafter, cells were washed with PBS and stained with crystal violet (15 min, 0.1% cristal violet in 10% methanol). The plates were washed three times with PBS. The stain was eluted with a solution containing 50% ethanol and 0.1 M sodium citrate (pH 4.2). Using a 96-well plate reader, the OD values of the elution solutions were measured at 570 nm. The mean OD values were calculated from three wells (triplicates) for each experimental condition.

#### GST Pull-Down Assay and Western Blot Analysis

The GTPases pull down assays were performed in neutrophils and differentiated PLB-985 cells as previously described by us [32, 33]. Neutrophils (10^7^ cells per assay) or differentiated PLB-985 cells (2 × 10^7^ cells per assay) were lysed in a buffer composed of 50 mM Tris–HCl, pH 7.5, 1% Triton X-100, 100 mM NaCl, 10 mM MgCl_2_, 5% glycerol, 1 mM Na_3_VO_4_, and a protease inhibitor tablet. The GST-RalGDS-RBD or the GST-PAKcrib fusion proteins were coupled to glutathione-sepharose beads for 1 h, and then the beads were added to the clarified cell lysates. After 1 h, the beads were collected by centrifugation and washed three times with lysis buffer. The beads were re-suspended in 2 × Laemmli sample buffer and boiled under reducing conditions. The proteins were subjected to 12% SDS-PAGE and transferred to polyscreen PVDF membranes. The membranes were blocked in Tris-buffered saline (TBS) supplemented with 0.2% Tween 20 (TBS/Tween) and 5% skimmed milk, and then incubated for 1 h overnight in a cold room with either anti-Rap1A/Rap1B mAb (1/2000 dilution), anti-Rac1/3 mAbs (1/2000 dilution) or anti-Cdc42 mAbs (1/2000 dilution). Antibodies were diluted in TBS/Tween 20. Thereafter, the membranes were washed in TBS/Tween 20, and subsequently incubated for 1 h with peroxidase-conjugated anti-rabbit IgGs (1:10000) in TBS/Tween 20. Antibody binding was visualized by enhanced chemiluminescence (ECL) and detection of emitted light using a G-box.

#### Determination of Histamine Concentration

Quantification of histamine in the modified HBSS medium was carried out using an ELISA kit by following the protocol and instructions provided by the manufacturer. Concentration of histamine was calculated by referring to a standard curve using Sigma plot.

#### Degranulation Assays

Neutrophils (1 × 10^6^) were incubated at 37 °C on 24-well plates coated with fibrinogen in the absence or presence of fMLP (1 μM), histamine (0.01–1 μM), or a combination of fMLP (1 μM) and histamine (0.01–1 μM). After 30 min incubation, the media were collected and the samples were subjected to low-speed centrifugation (190 g, 5 min). The supernatants, free of cells, were collected. The concentration of lactoferrin in the supernatants was determined using an ELISA as described by us [29]. The concentration of lactoferrin in each well was calculated based on the human lactoferrin calibration curve. For each experimental condition, the mean concentration of lactoferrin was calculated from 3 wells. In parallel, the enzymatic activities of neutrophil elastase (NE) [34, 35] in the supernatants were determined by measuring emission of fluorescence subsequently to the addition of a specific substrate of the enzyme.

#### Intracellular Calcium Measurement

Neutrophils were loaded with Fluo-4, a fluorescent dye for quantifying cellular Ca^2+^ concentrations. It is well exited at a wavelength of 488 nm. Calcium responses are expressed as F/F0 ratio [36].

#### Statistical Analysis

To compare the means of multiple groups (control versus treatments), we used the analysis of variance (ANOVA) statistical test. We assessed whether the *p*-value corresponding to the F-statistic test of one-way ANOVA was lower than 0.05 to evaluate if one or more pairs of groups were significantly different. When it was the case, we used a Tukey HSD test and a Scheffé multiple comparison test to identify which of the pairs of treatments are significantly different from each other. We also compared treatment groups relative to controls simultaneously by using Bonferroni correction to adjust probability values. An unpaired Student’s T-test was used to compare specific granule versus azurophil granule exocytosis and fold-increases of Rap1-, Rac-, and Cdc42- GTP levels in histamine stimulated cells versus control cells.

## Results

### Neutrophils Produce Histamine in Response to β2 Integrin-Dependent Adhesion

**Fig. 1:**
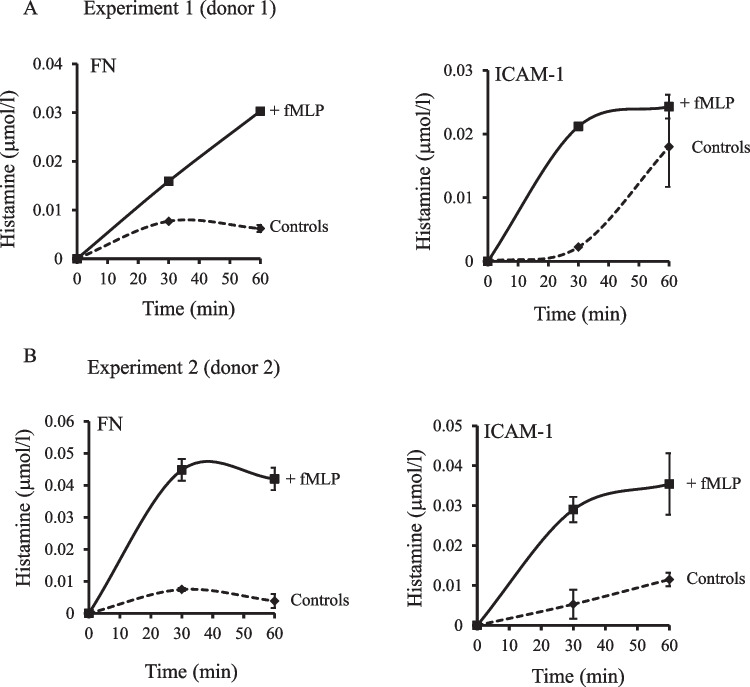
Histamine is produced by neutrophils adherent to β2 integrin ligands. Human neutrophils were incubated on 12-well plates coated with fibrinogen (FN) or ICAM-1 in the absence (controls) or presence of fMLP (10^–6^ M). After 30–60 min, the supernatants were collected. The concentration of histamine in the cell-free supernatants was measured by ELISA. The data represent mean values of triplicates ± SD of two representative experiments (**A** & **B**)

Unstimulated neutrophils (controls) incubated on plates coated with fibrinogen, a ligand for Mac-1, but not for LFA-1 [37], synthesized and released histamine in the medium. Basal histamine concentrations peaked at 30 min and remained unchanged over a longer incubation time. In experiment 1 and 2, maximal histamine concentrations reached ~ 7.7 nM (Fig. 1A, left panel) and 8.6 nM (Fig. 1B, left panel), respectively.

**Fig. 2:**
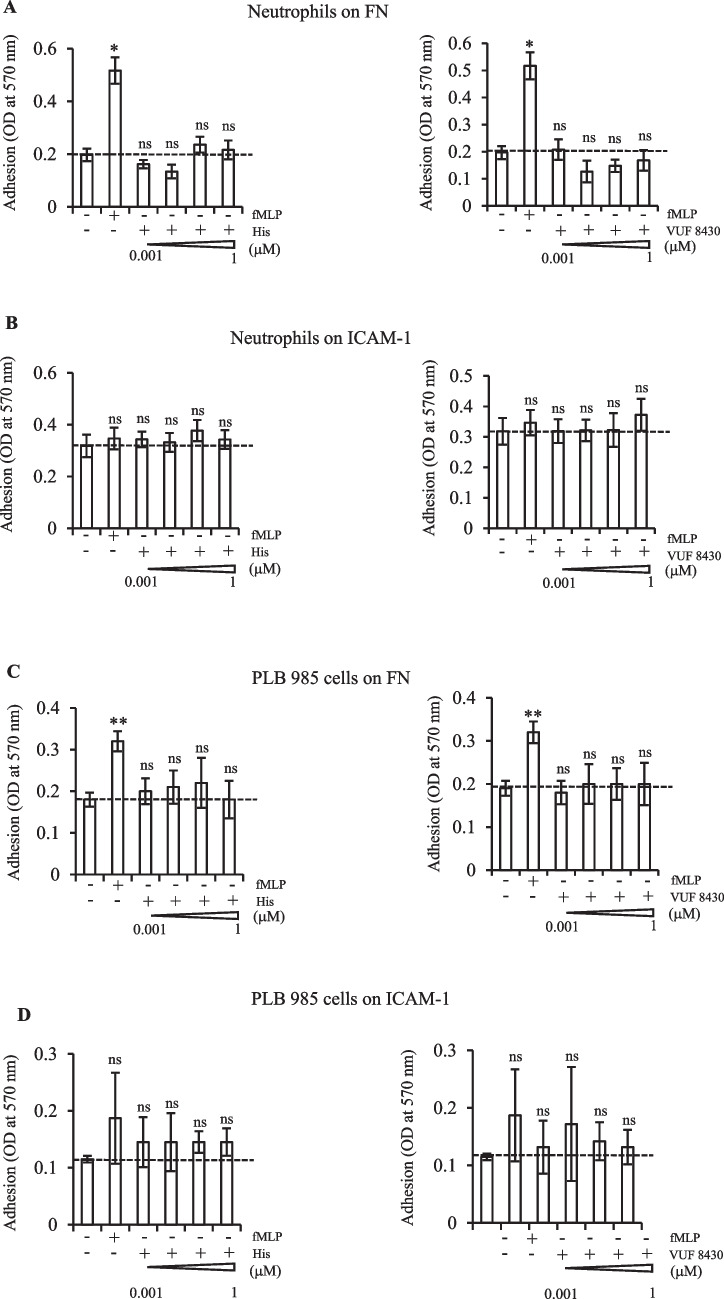
Histamine or the H_4_R agonist VUF 8430 did not promote neutrophil adhesion on β2 integrin ligands. **A** neutrophils (10^5^/well) were incubated on to 96-well plates coated with fibrinogen (FN) in the absence (-) or presence of histamine (0.001–1 μM) (left panel), or VUF 8430 (0.001–1 μM) (right panel). Adhesion in response to fMLP (1 μM) was used as a positive control. After 30 min, the supernatants were removed by aspiration, the wells were washed with PBS, and the relative amounts of adhered cells were determined by crystal violet staining as described in the Materials and Methods section. The data represent mean values of triplicates ± SD of 5 separate experiments. **B** experiments similar to the ones described in the section A above were performed with neutrophils incubated on to 96-well plates coated with ICAM-1. The data represent mean values of triplicates ± SD of 7 separate experiments. **C** neutrophil-like PLB-985 cells were incubated on to 96-well plates coated with fibrinogen after which adhesion in response to histamine (0.001–1 μM) (left panel) or VUF 8430 (0.001–1 μM) (right panel) were measured as described in the section A above. The data represent mean values of triplicates ± SD of 5–10 (A, left panel) or 4–10 (B, right panel) separate experiments. **D** experiments similar to the ones described in the section C above were performed with neutrophil-like PLB-985 cells incubated to 96-well plates coated with ICAM-1. The data represent mean values of triplicates ± SD of 4 separate experiments. Statistical analysis: Tukey’s multiple comparison test; *P < 0.05, **P < 0.01; ns, not significant

Addition of fMLP (1 μM) to neutrophils incubated on fibrinogen-coated wells increased basal histamine concentration in the medium (Fig. 1A and B, left panels) and neutrophil adhesion (Fig. 2A). Histamine concentrations reached ~ 30 nM (3.9-fold increase versus controls, Fig. 1A, left panel) and 45 nM (5.2- fold increase versus controls, Fig. 1B, left panel) at the 60 min incubation time point, in experiment 1 and 2, respectively.

We observed a different kinetic of histamine production by unstimulated neutrophils incubated to ICAM-1 (CD54), a ligand for both LFA-1 and Mac-1 [38]. After a lag phase (30 min) during which histamine in the medium was barely detectable, the concentration of histamine in the milieu rose. Addition of fMLP (1 μM) accelerated histamine production by adherent neutrophils reaching 24 nM (Fig. 1A, right panel, experiment 1) and 35 nM (Fig. 1B, right panel, experiment 2) at the 60 min incubation time, respectively. These concentrations of histamine activate the H_4_R but not the H_2_R [25]. Therefore, we next investigated whether the H_4_R played a role in neutrophil adhesion.

### Effect of Histamine and VUF 8430 on Neutrophil Adhesion on β2 Integrin Ligands

Neutrophils were incubated on plates coated with fibrinogen in the absence or presence of fMLP (1 μM), histamine (0.001–1 μM) or the H_4_R agonist VUF 8430 (0.001–1 μM). fMLP augmented by ~ 2.6-fold basal adhesion of neutrophils to fibrinogen (*P* < 0.01). In contrast, histamine or VUF 8430 did not (Fig. 2A). fMLP (1 μM), histamine (0.001–1 μM) or VUF 8430 (0.001–1 μM) did not augment neutrophil adhesion to ICAM-1-coated wells (Fig. 2B).

Similarly to what we found with neutrophils, fMLP augmented by ~ 1.7- fold (*P* < 0.01) basal adhesion of neutrophil-like PLB-985 cells to fibrinogen. In contrast, histamine (0.001–1 μM) or VUF 8430 (0.001–1 μM) did not (Fig. 2C). In addition, fMLP (1 μM), histamine (0.001–1 μM) or VUF 8430 (0.001–1 μM) did not augment basal adhesion of neutrophil-like PLB 985 cells to ICAM-1-coated wells (Fig. 2D).

### Histamine and VUF 8430 Potentiated fMLP-induced Neutrophil Adhesion to ICAM-1, But Not to Fibrinogen

**Fig. 3:**
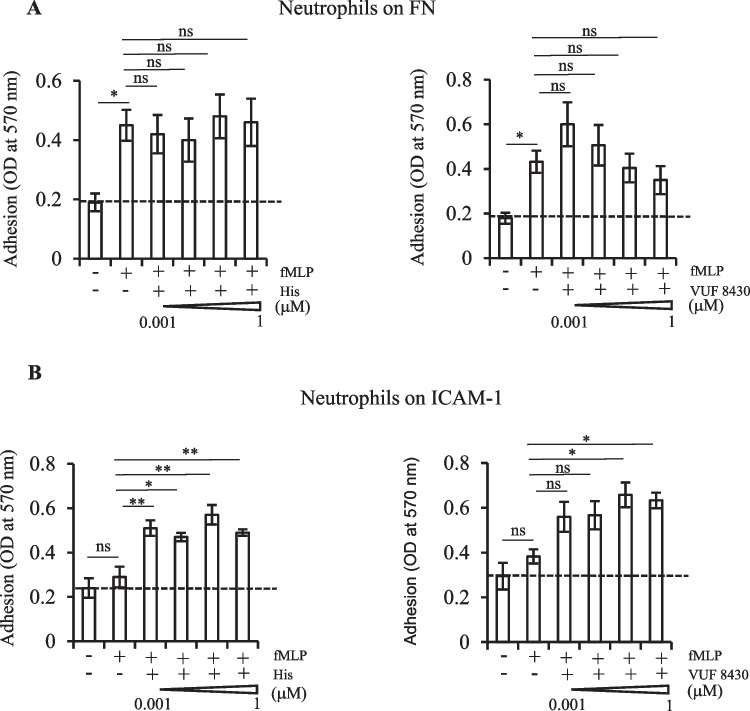
Effect of histamine and VUF 8430 on fMLP-induced neutrophil adhesion to β2 integrin ligands. **A** neutrophils (10^5^/well) were incubated on to 96-well plates coated with fibrinogen (FN) in the absence (-) or presence of fMLP (10^–6^ M) or a combination of fMLP (10^–6^ M) and histamine (0.001–1 μM) (left panel), or a combination of fMLP and VUF 8430 (0.001–1 μM) (right panel). After 30 min, the relative amounts of adhered cells were determined as described in the legend of Fig. 2. The data represent mean values of triplicates ± SD of 6 (A, left panel) or 6–7 (A, right panel) separate experiments. **B** neutrophils (10^5^/well) were incubated to 96-well plates coated with ICAM-1 in the absence (-) or presence of fMLP (10^–6^ M) or a combination of fMLP (10^–6^ M) and histamine (0.001–1 μM) (left panel), or a combination of fMLP and VUF 8430 (0.001–1 μM) (right panel). After 30 min, the relative amounts of adhered cells were determined as described in the section A above. The data represent mean values of triplicates ± SD of 5–6 (A, left panel) or 4–5 (A, right panel) separate experiments. Statistical analysis: Tukey’s multiple comparison test; *P < 0.05, **P < 0.01; ns, not significant

When histamine (10^–9^—10^–6^ M) or VUF 8430 (10^–9^—10^–6^ M) were added in combination with fMLP (10^–6^ M), the level of neutrophil adhesion to fibrinogen was similar to the level of adhesion measured in response to fMLP (10^–6^ M) alone (Fig. 3A).

Interestingly, neutrophil adhesion to ICAM-1-coated wells was not augmented in response to fMLP (Fig. 3B) but neutrophil adhesion was augmented in response to a combination of fMLP (10^–6^ M) and histamine (10^–9^ M—10^–6^ M). Notably, a concentration of histamine as low as 10^–9^ M, in combination with fMLP (10^–6^ M), was sufficient to augment by ~ 2.1-fold basal adhesion to ICAM-1 (*P* < 0.01) (Fig. 3B, left panel). A similar result was obtained with VUF 8430 (10^–7^−10^–6^ M) which augmented by ~ 2.2-fold (*P* < 0.05) basal neutrophil adhesion to ICAM-1 when combined with fMLP (10^–6^ M) (Fig. 3B, right panel).

### Histamine and Calcium Signaling in Neutrophils

**Fig. 4:**
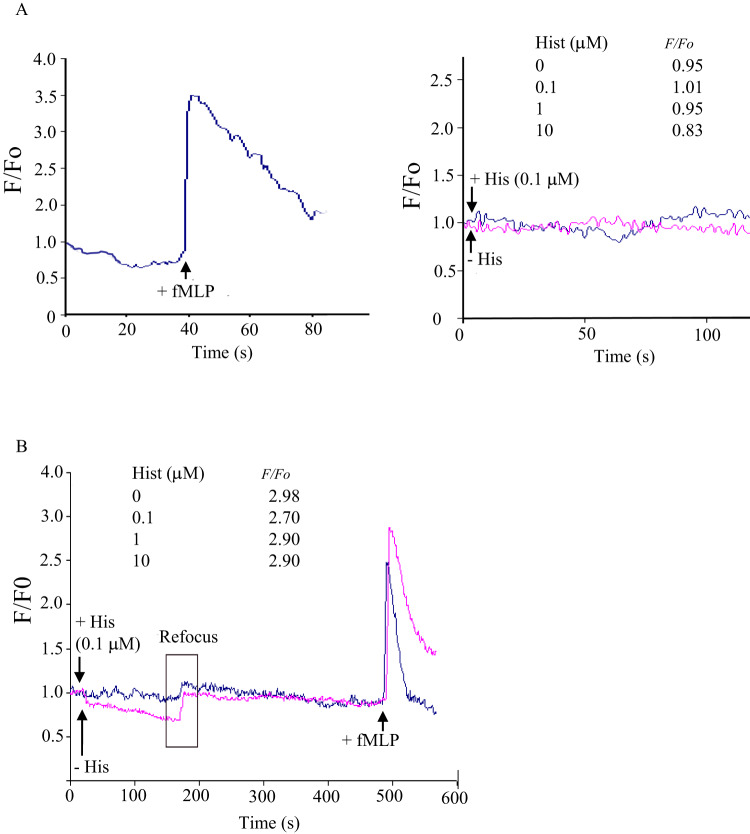
Histamine does not induce a calcium signal in neutrophils. Neutrophils were loaded with Fluo-4, after which the cells were stimulated with fMLP (A, left panel) or histamine (His, 0.1–10 μM) (A, right panel). The F/Fo ratio are shown on the y axis. The resting Fluoro-4 signal F/Fo = 1 was set by calculation. The F/Fo values obtained for each concentration of histamine used are shown. In B, histamine (His, 0.1 μM) was added or not (- His) to the medium containing neutrophils prior to the addition of fMLP. The F/Fo ratio was measured over time. The F/Fo values obtained for each concentration of histamine are shown

fMLP induces the expression of Mac-1 on the membrane surface of neutrophils [2] and promotes Mac-1-dependent adhesion (Fig. 2A). These processes are dependent on fMLP-induced elevation of intracellular calcium [Ca^2+^]_i_ [2]. We found that fMLP, but not histamine (0.1–10 μM), increased [Ca^2+^]_i_ in Fluoro-4-loaded neutrophils (Fig. 4A). A pretreatment of neutrophils with histamine (0.1–10 μM) had no effect on fMLP-induced increase in [Ca^2+^]_i_ (Fig. 4B).

### Histamine Activates Rap1 in a H_4_R-Dependent Manner

In neutrophils, activation of monomeric GTPases by histamine has not yet been reported. To investigate activation of Rap1 by histamine, neutrophil-like PLB-985 cells were stimulated with histamine for different time periods (0–120 s), after which the cells were lysed and GTP-bound Rap1 was precipitated using the GST-RalGDS fusion protein. The relative amounts of precipitated Rap1-GTP were assessed by Western blot analysis using anti-Rap1A/1B mAbs [33]. The rationale for using neutrophil-like PLB-985 cells is that these cells respond similarly to human neutrophils in terms of β2 integrin-dependent adhesion and activation of Rap1 and Rho family GTPases [32, 33].

**Fig. 5:**
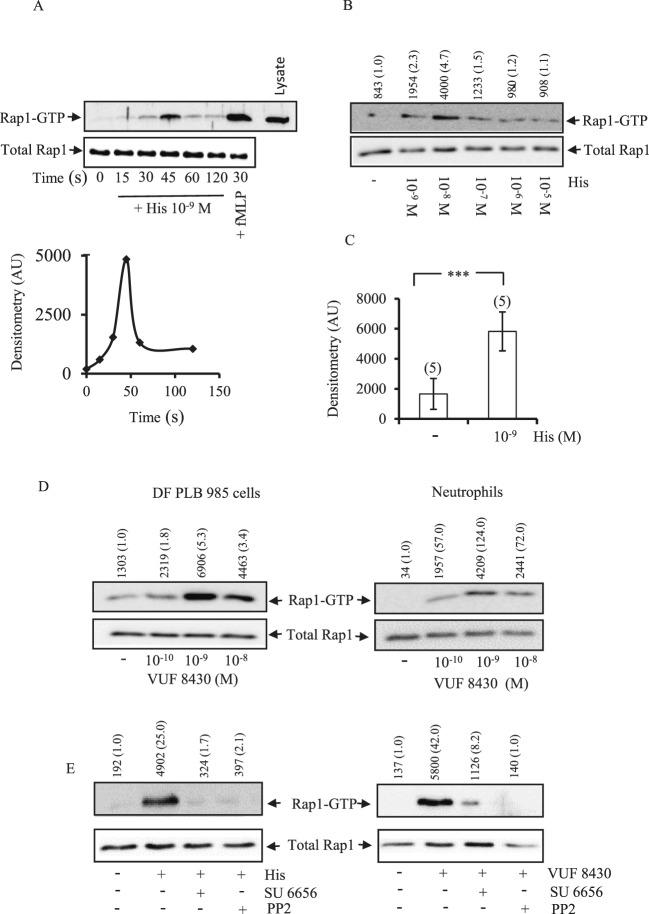
Histamine and VUF 8430 activate Rap1 in neutrophils and neutrophil-like PLB-985 cells. **A** neutrophil-like PLB-985 cells (20 × 10^6^) were stimulated for different time periods (0–120 s) with histamine (10^–9^ M) or fMLP (10^–7^ M, 60 s) after which the cells were lysed. GTP-bound Rap1 was pulled down using the GST-RalDGS fusion protein coupled to glutathione-sepharose beads. The amount of precipitated Rap1 was quantified by Western blot analysis using anti-Rap1A/Rap1B mAbs. A representative Western blot (out of 3) is shown in the top panel. The position of Rap1 is indicated by arrows on the left-hand side of the panel. In the bottom panel, densitometry analysis of the Western blot is shown and illustrate changes of GTP-bound Rap1 levels over time. **B** neutrophil-like PLB-985 cells (20 × 10^6^) were stimulated for 1 min with different concentrations of histamine (His) (10^–9^ M- 10^–5^ M) after which the cells were lysed and the levels of GTP-bound Rap1 were measured as described in section A above. The densitometric values are shown in the top of the blot. The amount of GTP-bound Rap1 in control cells has been normalized to one. A representative experiment (out of 3) is shown. **C** quantification of the level of GTP-bound Rap1 in neutrophil-like cells stimulated with histamine (10^–9^ M, 45 s). The data are expressed as mean values ± SD of 5 independent experiments. ***P < 0.001. **D** neutrophil-like PLB-985 cells (left panel), or neutrophils (right panel) were incubated with VUF 8430 (10^–10^ M- 10^–8^ M) for 1 min after which the cells were lysed and the amounts of GTP-bound Rap1 were measured as described in the sections A-B above. The densitometric values are shown in the top of the blot. The amount of Rap1-GTP in control cells has been normalized to one. A representative experiment (out of 3) is shown. **E** neutrophil-like PLB-985 cells were pre-incubated with the Src family tyrosine kinase inhibitors SU6656 (5 μM) or PP2 (5 μM) for 20 min after which histamine (10^–8^ M) (left panel) or VUF 8430 (10^–9^ M) (right panel) were added for 1 min. Thereafter, the cells were lysed and the amount of GTP-bound Rap1 was measured as described in the sections above. The densitometric values are shown in the top of the blots. The amount of GTP-bound Rap1 in control cells has been normalized to one. A representative experiment (out of 3) is shown

We found that incubation of neutrophil-like PLB-985 cells with a concentration of histamine as low as 10^–9^ M led to a transient activation of Rap1 (Fig. 5A). Maximum activation of Rap1 was obtained after 45 s stimulation (14-fold increase over time 0). Histamine (10^–9^—10^–5^ M) induced, over a one-minute time period, a dose-dependent activation of Rap1 (Fig. 5B). Maximum activation of Rap1 was obtained with a dose of 10^–8^ M histamine. We could not detect significant activation of Rap1 with high concentrations of histamine (10^–6^ M—10^–5^ M). When we combined the data of Rap1 activation in response to histamine from several independent experiments, we found a 3.5-fold increase in Rap1 activation in cells stimulated for 1 min with 10^–9^ M histamine (*P* < 0.001) (Fig. 5C).

We confirmed that the H_4_R was involved in the activation of Rap1 by showing that incubation of neutrophil-like PLB-985 cells or neutrophils with the H_4_R agonist VUF 8430 (10^–10^—10^–8^ M) augmented in a dose-dependent manner the GTP-loading of Rap1 (Fig. 5D).

A pre-treatment of neutrophil-like PLB 985 cells with the Src family tyrosine kinase inhibitors PP2 (5 μM) or SU 6656 (5 μM) totally abolished histamine- or VUF 8430—induced activation of Rap1 (Fig. 5E).

### Histamine Activates Rac GTPases in a H_4_R-dependent Manner

To investigate the regulation of Rac GTPases in response to histamine, we used the GST-PakCrib fusion protein to pull-down the GTP-bound forms of Rac from cells, stimulated or not with histamine, followed by Western blot analysis using anti-Rac1/3 antibodies [31].

**Fig. 6:**
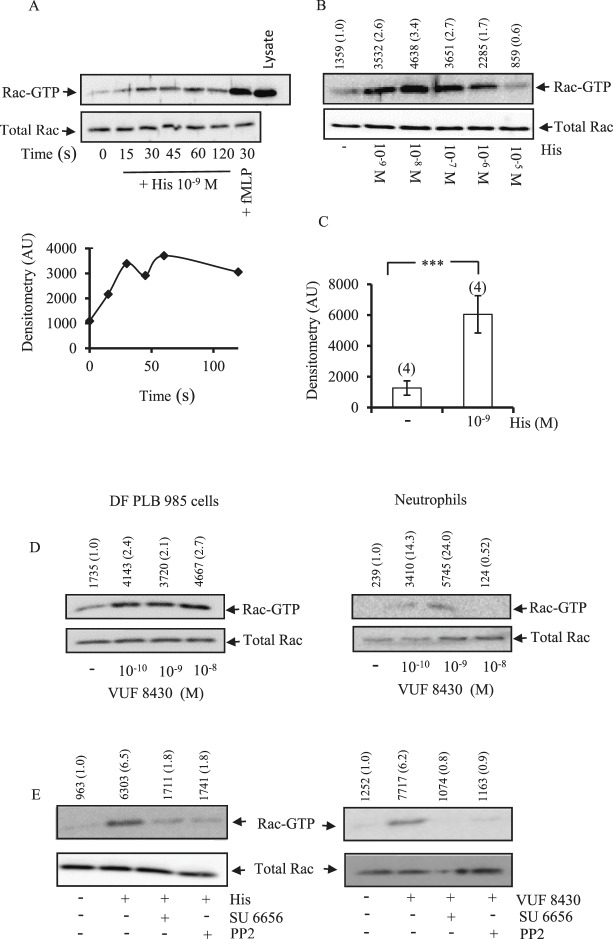
Histamine and VUF 8430 activate Rac1/2 in neutrophils neutrophil-like PLB-985 cells. **A** neutrophil-like PLB-985 cells (20 × 10^6^) were stimulated for different time periods (0–120 s) with histamine (10^–9^ M) or fMLP (10^–7^ M, 60 s) after which the cells were lysed. Levels of GTP-bound Rac was measured using the GST-PAK crib fusion protein coupled to glutathione Sepharose beads followed by Western blot analysis using anti- Rac1/3 mAbs. A representative Western blot (out of 3) is shown in the top panel. The position of Rac is indicated by arrows. In the bottom panel, densitometry analysis of the Western blot is shown and illustrate changes of GTP-bound Rac levels over time. **B** neutrophil-like PLB-985 cells (20 × 10^6^) were stimulated for 1 min with different concentrations of histamine (His) (10^–9^ M- 10^–5^ M) after which the cells were lysed and the levels of GTP-bound Rac were measured as described in section A above. The densitometric values are shown in the top of the blots. The amount of Rac-GTP in control cells has been normalized to one. A representative experiment (out of three) is shown. **C** quantification of the level of GTP-bound Rac in neutrophil-like cells stimulated with histamine (10 ^9^ M, 45 s). The data are expressed as mean values ± SD of 4 independent experiments. ***P < 0.001. **D** neutrophil-like PLB-985 cells (left panel), or neutrophils (right panel) were incubated with VUF 8430 (10^–10^ M- 10^–8^ M) for 1 min after which the cells were lysed and the amounts of GTP-bound Rac were measured as described in the sections A-B above. The densitometric values are shown in the top of the blots. The amount of Rac-GTP in control cells has been normalized to one. A representative experiment (out of three) is shown. **E** neutrophil-like PLB-985 cells were pre-incubated with the Src family tyrosine kinase inhibitors SU6656 (5 μM) or PP2 (5 μM) for 20 min after which histamine (10^–8^ M) (left panel) or VUF 8430 (10^–9^ M) (right panel) were added for 1 min. Thereafter, the cells were lysed and the amounts of GTP-bound Rac were measured as described in the sections above. The densitometric values are shown in the top of the blot. The amount of Rac-GTP in control cells has been normalized to one. A representative experiment (out of three) is shown

We found that a nanomolar concentration of histamine (10^–9^ M) induced a sustained activation of Rac GTPases in neutrophil-like PLB-985 cells (Fig. 6A). Maximum GTP-loading of Rac GTPases was observed after 30 s stimulation with histamine (3.5-fold increase over time 0). Histamine (10^–9^ M −10^–6^ M) augmented in a dose-dependent manner the levels of Rac-GTP in neutrophil-like PLB-985 cells over a 1 min incubation time (Fig. 6B). Maximum GTP-loading of Rac was obtained with 10^–8^ M histamine (~ threefold increase over controls). When we combined the data of Rac activation in response to histamine from several independent experiments, we found a 4.8-fold increase in Rac activation in cells stimulated for 1 min with 10^–9^ M histamine (*P* < 0.001) (Fig. 6C).

VUF 8430 (10^–10^ M −10^–8^ M) induced a dose-dependent activation of Rac GTPases in neutrophil-like PLB-985 cells (Fig. 6D, left panel) and neutrophils (Fig. 6D, right panel).

The Src family tyrosine kinases PP2 (5 μM) or SU 6656 (5 μM) abolished Rac activation induced by histamine (10 ^−9^ M) (Fig. 6E, left panel) or VUF 8430 (10^–9^ M) (Fig. 6E, right panel).

### Histamine Activates Cdc42 in a H_4_R-dependent Manner

**Fig. 7:**
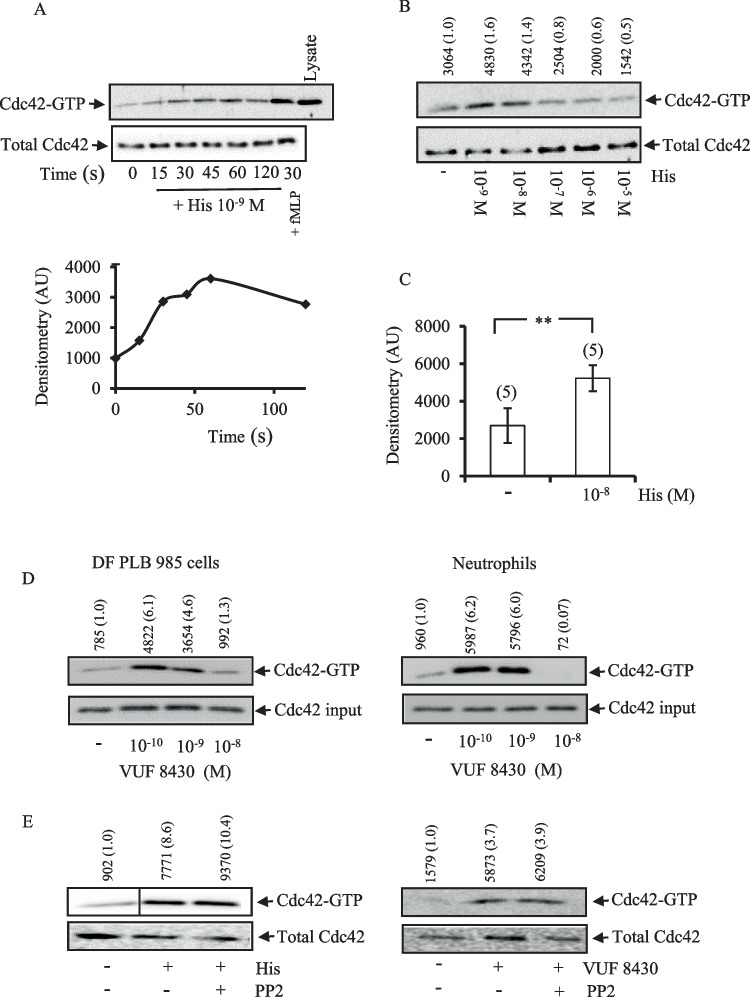
Histamine and VUF 8430 activate Cdc42 in neutrophils and neutrophil-like PLB-985 cells. **A** neutrophil-like PLB-985 cells (20 × 10^6^) were stimulated for different time periods (0–120 s) with histamine (10^–9^ M) or fMLP (10^–7^ M, 60 s) after which the cells were lysed. Levels of GTP-bound Cdc42 were measured using the GST-PAK crib fusion protein followed by Western blot analysis using an anti-Cdc42 mAbs. A representative Western blot (out of 3) is shown in the top panel. The position of Cdc42 is indicated by arrows on the left-hand side of the panel. In the bottom panel, densitometry analysis of the Western blot is shown and illustrates changes of Cdc42-GTP levels over time. **B** neutrophil-like PLB-985 cells (20 × 10^6^) were stimulated for 1 min with different concentrations of histamine (His) (10^–9^ M—10^–5^ M) after which the cells were lysed and the levels of GTP-bound Cdc42 were measured as described in section A above. The densitometric values are shown in the top of the blot. The amount of Cdc42-GTP in control cells has been normalized to one. A representative experiment (out of three) is shown. **C** quantification of the level of GTP-bound Cdc42 in neutrophil-like cells stimulated with histamine (10^–8^ M, 1 min). The data are expressed as mean values ± SD of 4 independent experiments. ***P < 0.001. **D** neutrophil-like PLB-985 cells (left panel), or neutrophils (right panel) were incubated with VUF 8430 (10^–10^ M- 10^–8^ M) for 1 min after which the cells were lysed and the amounts of GTP-bound Cdc42 were measured as described in the sections A-C above. The densitometric values are shown in the top of the blots. The amount of Cdc42-GTP in control cells has been normalized to one. A representative experiment (out of three) is shown. **E** neutrophil-like PLB-985 cells were pre-incubated with the Src family tyrosine kinase PP2 (5 μM) for 20 min after which histamine (10^–8^ M) (left panel) or VUF 8430 (10^–9^ M) (right panel) were added for 1 min. Thereafter, the cells were lysed and the amounts of GTP-bound Cdc42 were measured as described in the sections above. The densitometric values are shown in the top of the blot. The amount of Cdc42-GTP in control cells has been normalized to one. A representative experiment (out of three) is shown

The GST-PakCrib fusion protein can also be used to pull down the GTP-bound form of Cdc42. Histamine (10^–9^ M) activated in a time-dependent manner Cdc42 in neutrophil-like PLB-985 cells. Maximum Cdc42-GTP levels were found after 45–60 s stimulation with histamine (3.5-fold increase over time 0) and remained sustained over time (Fig. 7A). Histamine (10^–9^ M—10^–5^ M) induced a dose-dependent activation of Cdc42 (Fig. 7B). In Fig. 7C, levels of GTP-bound Cdc42 were expressed as mean values from several different experiments. Histamine (10^–8^ M, 1 min) induced a 1.9-fold increase of Cdc42 activity over unstimulated control cells (*P* < 0.05). VUF 8430 (10^–10^ M- 10^–8^ M) augmented activation of Cdc42 in neutrophil-like PLB-985 cells (Fig. 7D, left panel) and neutrophils (Fig. 7D, right panel). In contrast to what we found with Rap1 and Rac GTPases, a pretreatment of neutrophil-like PLB-985 cells with PP2 did not prevent activation of Cdc42 by histamine (10^–9^ M) or VUF 8430 (10^–9^ M) (Fig. 7E).

### Role of Histamine in Adherent-dependent Neutrophil Degranulation

**Fig. 8:**
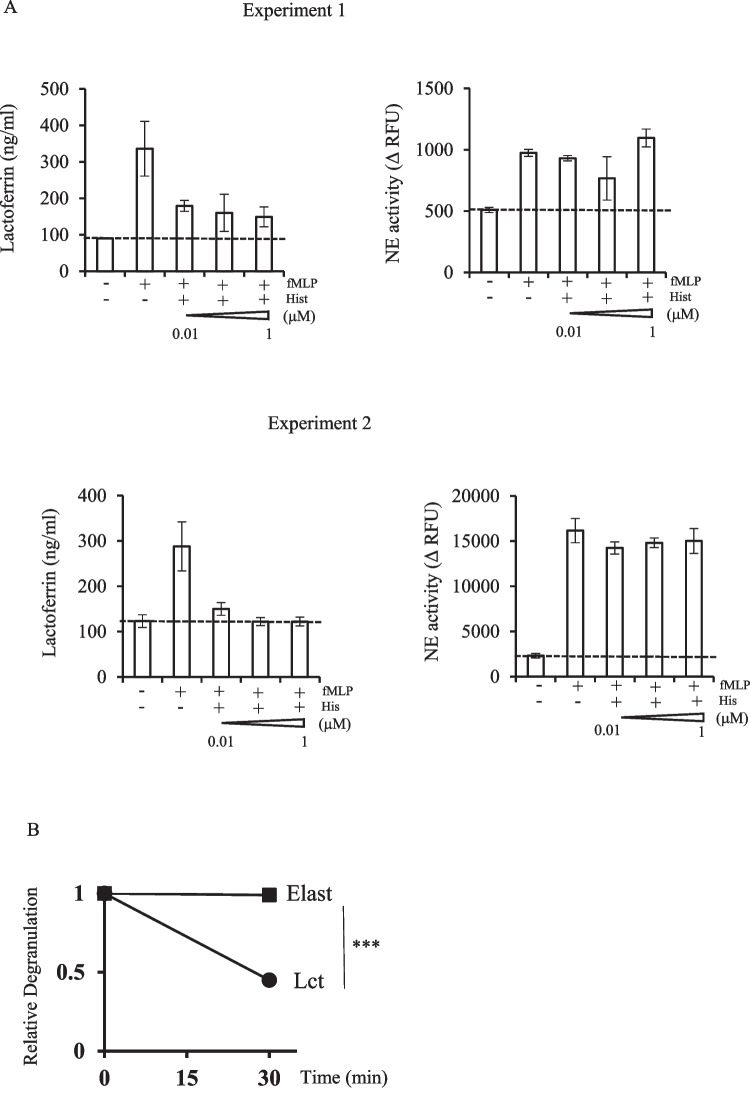
Histamine inhibits neutrophil adhesion-dependent specific granule exocytosis but not azurophil granule exocytosis. **A** neutrophils (10^6^) were incubated on plates coated with fibrinogen in the absence (-) or presence of fMLP (10^–6^ M), or fMLP (10^–6^ M) in combination with histamine (0.01–1 μM). After 30 min incubation at 37.^o^ C, the supernatants were collected, and the concentration of lactoferrin (left panels) or the enzymatic activity of neutrophil elastase (NE) (right panels) in the cell-free supernatants were measured as described in the Materials and Methods section. The data represent mean values of triplicates ± SD for each experiment. **B** the relative exocytosis of lactoferrin (Lct) versus elastase (Elast) was measured in samples obtained from neutrophils isolated from a larger cohort of donors (*n* = 4–5). Degranulation values at time 30 min were normalized to degranulation values at time 0. ***P < 0.001

We compared the effect of histamine on specific granule and azurophil granule exocytosis in neutrophils adherent to fibrinogen-coated wells. Incubation of neutrophils on plates coated with fibrinogen exhibited exocytosis of specific granules (lactoferrin release) and azurophil granules (release of elastase) in response to fMLP (Fig. 8A). Histamine (10^–8^ M −10^–6^ M) inhibited the release of lactoferrin by adherent neutrophils stimulated with fMLP (Fig. 8A, left panels). In contrast, the diamine (10^–8^ M −10^–6^ M) had no effect on the release of elastase (Elast) by adherent neutrophils stimulated with fMLP (Fig. 8A, right panels).

We next compared the effect of histamine on the release of lactoferrin (specific granule marker), and elastase (azurophil granules marker) in neutrophils prepared from a larger cohort of blood donors. Histamine inhibited the release of lactoferrin, but not elastase by neutrophils adherent to fibrinogen (*P* < 0.001, lactoferrin release versus elastase) (Fig. 8B). These results demonstrated that histamine selectively blocks specific granule but not azurophil granule exocytosis by adherent neutrophils.

## Discussion

Neutrophils produce the intermediary chemoattractants LTB_4_ [17, 18] and IL-8 [20] in response to end-target chemoattractants (C5a, formyl peptides) present at high concentrations at the site of infection. In this study, we provided evidence that neutrophils adherent on β2 integrin ligands, in response to fMLP, synthesized and released histamine. Histamine production by neutrophils incubated on ICAM-1- coated wells augmented markedly with time. These data may be explained by the fact that unstimulated neutrophils exhibit LFA-1-dependent, but not Mac-1- dependent, attachment to ICAM-1 and outside-in signaling [39]. Mac-1- or LFA-1- mediated histamine production was accelerated in response to fMLP and peaked between 30–60 incubation time. A similar time frame for histamine production was observed in neutrophils isolated from allergic individuals exposed in vitro to allergens and this was achieved through cross-linking of neutrophil IgE receptors (FCεRI, FcRII/CD23) [40].

LFA-1 mediates leukocyte initial adhesion to the vascular endothelium and extravasation [41]. ICAM-1 is the most effective ligand for mediating such adhesion due to the high bond strength of LFA-1 and ICAM-1 [42]. Mac-1 is required for intraluminal crawling, and adhesion while the leukocytes migrate through the interstitial space [41].

Histamine augments expression of Mac-1 on the membrane surface of eosinophils, modifies the shape of these cells by regulating actin polymerization and intracellular calcium levels and induces eosinophil chemotaxis [43, 44]. These effects of histamine in eosinophils are mediated via the H_4_R as they were blocked by the H_3_R/H_4_R antagonist thiopeperamide or the H_4_R antagonist JNJ 7777120 [43, 44]. In contrast, histamine did not induce Mac-1-dependent neutrophil adhesion and cell spreading [29], or neutrophil shape change [43, 44]. The fact that fMLP, but not histamine, augmented [Ca2^+^]_i_, a prerequisite for Mac-1 expression on the cell surface, may explain, why only fMLP increased Mac-1-dependent neutrophil adhesion. Leino et al. [45] found that histamine inhibited fMLP-induced rise in [Ca^2+^]_i_ in neutrophils but no information was provided on whether histamine inhibited fMLP-induced Ca^2+^-dependent neutrophil functions. In contrast, we found that histamine had no effect on the rise of [Ca^2+^]_i_ induced by fMLP. This finding is in keeping with our data showing that histamine did not modify fMLP-induced Mac-1- and Ca^2+^- dependent neutrophil adhesion.

Mac-1 and LFA-1 are the dominant integrins controlling chemotaxis to fMLP and the intermediary chemoattractant IL-8, respectively [46]. Since histamine did not regulate Mac-1-dependent neutrophil adhesion (a hallmark of primary chemoattractants), we investigated whether histamine shared similar features to intermediary ligands which regulate LFA-1-dependent neutrophil adhesion and functions. To this end, we tested the ability of histamine to induce neutrophil adhesion to ICAM-1. ICAM-1 is a ligand for Mac-1 and LFA-1, but when both LFA-1 and Mac-1 are expressed in leukocytes, cell adhesion is predominantly mediated by LFA-1 [41] because LFA-1 has a greater avidity for ICAM-1 than does Mac-1 [42]. Indeed, Mac-1-dependent neutrophil adhesion to ICAM-1 was only observed in neutrophils isolated from LFA-1^−/−^ knock out mice [41].

We discovered that histamine or VUF 8430 did not induce LFA-1- dependent neutrophil adhesion to ICAM-1. Likewise, fMLP did not augment mouse neutrophil adhesion to ICAM-1 and this was because all resting neutrophils adhered to ICAM-1-coated wells and therefore fMLP could not further augment the number of adherent neutrophils [42]. Other investigators found that in resting peripheral blood lymphocytes, LFA-1 was inactive and weakly bound to ICAM-1, and no increase in adhesion was observed following PMA stimulation [5]. Interestingly, fMLP up-regulates expression of Mac-1 [2], but not LFA-1 [42], on the neutrophil membrane surface and augments the expression of the LFA-1 activation epitopes mAb24 [29], KIM-127, and NKI-L16 [47]. These findings may indicate that conformational activation of LFA-1 is not sufficient to establish a strong LFA-1/ICAM-1 bond and adhesion to the vascular endothelium.

Evidence for a change in LFA-1 avidity for the regulation of LFA-1-dependent leukocyte adhesion and chemotaxis has been documented. First, LFA-1 clustering and LFA-1-dependent neutrophil migration are minimally activated by fMLP [46]. Second, the intermediary chemoattractant IL-8 activates LFA-1 clustering, but not LFA-1 membrane surface expression, and controls directional movement of cells [46]. Third, in lymphocytes, increased binding activity to endothelial ligands is largely mediated by changes in LFA-1 avidity [7, 8]. Fourth, by using the erythroleukemic cell line K562 expressing mutant LFA-1 cytoplasmic receptors, it was shown that LFA-1-mediated cell adhesion to ICAM-1 was predominantly regulated by receptor clustering through the temporary dissociation of the cytoplasmic tail of LFA-1 from the cytoskeleton [5].

We found that fMLP augmented basal adhesion of neutrophil to ICAM-1 only when combined with histamine. This finding implies that the inability of fMLP to augment neutrophil adhesion is not due to all resting neutrophils adhering to ICAM-1. The potentiation effect of histamine was mediated via the H_4_R. Indeed, a nanomolar concentration of histamine or VUF 8430, combined with fMLP, augmented basal neutrophil adhesion to ICAM-1. Thus, intracellular signaling pathways emanating from both receptors may converge to augment LFA-1-dependent neutrophil adhesion. Since fMLP induces conformational changes of LFA-1, but neither fMLP nor histamine augmented neutrophil adhesion to ICAM-1, it is tempting to propose that signals emanating from the H_4_R and the formyl peptide receptor 1 (FPR1) contribute to LFA-1 avidity change.

The FPR1 and the H_4_R are Gi protein-coupled receptors [48–50]. We showed that activation of these receptors in neutrophils, or neutrophil-like PLB-985 cells, by their respective ligands, led to a similar pattern of tyrosine phosphorylated proteins thus reflecting activation of Src family tyrosine kinases [24]. These enzymes are indispensable for neutrophil adhesion by β2 integrins and activation of inflammatory functions [51]. However, despite resemblances in terms of signaling, both receptors behave differently in terms of promoting neutrophil adhesion to Mac-1 indicating that these receptors also exhibit differences in terms of signaling as evidenced by differential mobilization of intracellular calcium.

It is intriguing that histamine regulates the adhesive and migration capacities of eosinophils [43, 44] but not neutrophils. Histamine is produced by neutrophils in response to bacterial and fungal infections [22, 23] and by mast cells in response to protozoans best exemplified by increased plasma levels of histamine and IgE anti-malarial antibodies in individuals with falciparum malaria [52]. During parasitic infections, eosinophils are the dominant leukocytes migrating to the site of infection. It is plausible that neutrophils can distinguish between these two different types of infection during which histamine is produced, to regulate accordingly their migration to inflamed tissues. Thus, during helminth infections, histamine produced by mast cells would attract dominantly eosinophils and minimally neutrophils because histamine cannot promote neutrophil adhesion to ICAM-1 without the concomitant presence of a soluble mediator of bacterial origin. The requirement for both histamine and a signature of bacterial infection may be a safeguard mechanism to prevent unwanted migration of neutrophils during episodes of helminth infections.

Activation of neutrophil inflammatory functions is largely dependent on Rho and Ras family members [53]. Many soluble mediators including fMLP, PAF and LTB_4_ induce GTP-loading of Rac, Cdc42, and Rap GTPases in neutrophils [54–57]. We now show that histamine activates Rap1, Rac1/2 and Cdc42 in neutrophils and neutrophil-like PLB-985 cells thus revealing that histamine produced by neutrophils regulates neutrophil functions.

Histamine and the H_4_R agonist VUF 8430 activated Rap1 in differentiated PLB-985 cells and neutrophils. Thus, the inability of histamine or VUF 8430 to promote neutrophil adhesion on β2 integrin ligands cannot be explained by a lack of Rap1 activation, which implies that activation of Rap1 is not sufficient to promote neutrophil adhesion on β2 integrin ligands.

Engagement of the H_4_R in neutrophils accelerated the killing of internalized complement- opsonised *E. coli* via a mechanism involving Src family tyrosine kinases [24]. This finding may be explained by the ability of the H_4_R to activate Rap1 and Rac1/2 which control fusion of granules containing antimicrobials with phagosomes (Rac1/2) and production of ROS (Rac1/2 and Rap1).

We previously showed that engagement of the H_4_R blocked specific granule exocytosis in neutrophils made adherent by β2 integrins [29]. Based on these findings, we proposed that the H_4_R had anti-inflammatory functions. However, we now discovered that engagement of the H_4_R led to activation of the small GTPases Rap1, Rac1/2 and Cdc42, which regulate neutrophil inflammatory functions. To interpret these seemingly contradictory results, we compared the effect of histamine on specific and azurophilic granule exocytosis. We found that histamine blocked fMLP-induced β2 integrin-dependent specific granule (lactoferrin release) but not azurophil granule (elastase release) exocytosis. Interestingly, histaminase, one of the enzyme catabolizing histamine is in specific granules [58]. Thus, inhibition by histamine of specific granule exocytosis by adherent neutrophils may be a mechanism to reduce histaminase activity in the extracellular milieu and tissues. This would lead to the gradual accumulation of histamine and activation of the H_4_R-mediated inflammatory response through regulation of LFA-1-dependent adhesion and activation of monomeric GTPases. When the concentration of histamine reaches the micromolar range, the H_4_R is switched off (desensitization) and the H_2_R is switched on leading to the resolution of the inflammatory response [24].

The fact that histamine does not block β2 integrin-induced exocytosis of azurophil granules, which are packed with acid hydrolases and antimicrobial proteins (MPO, elastase and defensins), ensures that the antimicrobial response remains sufficient to clear pathogens in the tissue environment or in phagosomes. More in depth studies will be performed in the future to investigate whether histamine regulates exocytosis of gelatinase B-containing granules (MMP-9 release) and to understand the mechanism by which histamine selectively inhibits neutrophil specific granule exocytosis.

In summary, we made novel findings on the role of the H_4_R in the regulation of neutrophil functions. First, we showed that histamine is produced by neutrophils adhering on β2 integrin ligands. Second, fMLP induced neutrophil adhesion to ICAM-1 only when the H_4_R is engaged. Third, engagement of the H_4_R led to GTP loading of Rap1, Rac1/2 and Cdc42 thus illustrating the key role played by the H_4_R in activation of neutrophil inflammatory functions. Fourth, engagement of the H_4_R led to inhibition of specific granule, but not azurophil granule, exocytosis to prevent the catabolism of histamine produced during periods of infection.

## Data Availability

All data supporting the findings of this study are available within the paper and its Supplementary Information.
